# Large-scale collection and annotation of full-length enriched cDNAs from a model halophyte, *Thellungiella halophila*

**DOI:** 10.1186/1471-2229-8-115

**Published:** 2008-11-12

**Authors:** Teruaki Taji, Tetsuya Sakurai, Keiichi Mochida, Atsushi Ishiwata, Atsushi Kurotani, Yasushi Totoki, Atsushi Toyoda, Yoshiyuki Sakaki, Motoaki Seki, Hirokazu Ono, Yoichi Sakata, Shigeo Tanaka, Kazuo Shinozaki

**Affiliations:** 1 Faculty of Applied Bioscience, Tokyo University of Agriculture, 1-1-1 Sakuragaoka, Setagaya-ku, Tokyo 156-8502, Japan; 2 Laboratory of Plant Molecular Biology, RIKEN Tsukuba Institute, 3-1-1 Koyadai, Tsukuba, Ibaraki 305-0074, Japan; 3 RIKEN Plant Science Center, 1-7-22 Suehiro-cho, Tsurumi-ku, Yokohama, Kanagawa 230-0045, Japan; 4 RIKEN Genomic Sciences Center, 1-7-22 Suehiro-cho, Tsurumi-ku, Yokohama, Kanagawa 230-0045, Japan; 5 MetaSystems Research Team, RIKEN Advanced Science Institute, Yokohama, 230-0045, Japan

## Abstract

**Background:**

*Thellungiella halophila *(also known as *Thellungiella salsuginea*) is a model halophyte with a small plant size, short life cycle, and small genome. It easily undergoes genetic transformation by the floral dipping method used with its close relative, *Arabidopsis thaliana*. *Thellungiella *genes exhibit high sequence identity (approximately 90% at the cDNA level) with Arabidopsis genes. Furthermore, *Thellungiella *not only shows tolerance to extreme salinity stress, but also to chilling, freezing, and ozone stress, supporting the use of *Thellungiella *as a good genomic resource in studies of abiotic stress tolerance.

**Results:**

We constructed a full-length enriched *Thellungiella *(Shan Dong ecotype) cDNA library from various tissues and whole plants subjected to environmental stresses, including high salinity, chilling, freezing, and abscisic acid treatment. We randomly selected about 20 000 clones and sequenced them from both ends to obtain a total of 35 171 sequences. CAP3 software was used to assemble the sequences and cluster them into 9569 nonredundant cDNA groups. We named these cDNAs "RTFL" (RIKEN *Thellungiella *Full-Length) cDNAs. Information on functional domains and Gene Ontology (GO) terms for the RTFL cDNAs were obtained using InterPro. The 8289 genes assigned to InterPro IDs were classified according to the GO terms using Plant GO Slim. Categorical comparison between the whole Arabidopsis genome and *Thellungiella *genes showing low identity to Arabidopsis genes revealed that the population of *Thellungiella *transport genes is approximately 1.5 times the size of the corresponding Arabidopsis genes. This suggests that these genes regulate a unique ion transportation system in *Thellungiella*.

**Conclusion:**

As the number of *Thellungiella halophila *(*Thellungiella salsuginea*) expressed sequence tags (ESTs) was 9388 in July 2008, the number of ESTs has increased to approximately four times the original value as a result of this effort. Our sequences will thus contribute to correct future annotation of the *Thellungiella *genome sequence. The full-length enriched cDNA clones will enable the construction of overexpressing mutant plants by introduction of the cDNAs driven by a constitutive promoter, the complementation of *Thellungiella *mutants, and the determination of promoter regions in the *Thellungiella *genome.

## Background

*Thellungiella halophila *(also known as *Thellungiella salsuginea*) is well known as a model halophyte for studying abiotic stress tolerance, as the plant exhibits extreme salt and freezing tolerance [1-9]. *Thellungiella *is closely related to Arabidopsis, and its genes share approximately 90% identity to those of Arabidopsis [1,10,11]. Moreover, *Thellungiella *is characterized by good features from the perspective of genetic studies, such as small plant size, a short life cycle, a high seed number, and the ability to self-pollinate. Furthermore, as in Arabidopsis, transformation of *Thellungiella *plants can be accomplished by means of the floral dipping method. Since the sequence identities between *Thellungiella *and Arabidopsis are very high at the cDNA level, Arabidopsis cDNA microarrays or oligo-microarrays can be used for transcriptome analysis of *Thellungiella *plants. We previously compared expression levels of various genes between *Thellungiella *and Arabidopsis plants under normal or high-salinity conditions using an Arabidopsis cDNA microarray composed of 7,000 Arabidopsis genes. Interestingly, a large number of genes known to be inducible by abiotic and biotic stresses were highly expressed in *Thellungiella *under normal growth conditions [5]. The use of a 70-mer oligoarray with 25 000 Arabidopsis genes revealed that Arabidopsis exhibited a global defense strategy required for bulk protein synthesis, whereas induced genes in *Thellungiella *were involved in protein folding, modification, and redistribution [2]. However, because of failed hybridization or a low hybridization rate between Arabidopsis DNAs and *Thellungiella *mRNAs, the data obtained from heterologous microarrays cannot provide an accurate evaluation of the expression levels. Recently, *Thellungiella *plants (Yukon ecotype) treated with drought, salinity, and freezing stresses were used to construct expressed sequence tag (EST) libraries with a total of 3628 unique genes [9]. A cDNA microarray was established with these cDNAs, and the transcriptional profiles of *Thellungiella *plants under various stress conditions were obtained [8].

Full-length cDNAs are useful genomic resources not only for genome annotation, but also for the identification of promoter regions, transgenic analyses, biochemical analyses, and determination of the three-dimensional structure of proteins [12]. Full-length enriched cDNA libraries from Arabidopsis [13,14], rice [15], poplar [16,17], wheat [18], maize [19], humans [20], mice [21,22], and *Drosophila *[23-25] have contributed enormously to elucidating biological processes in these organisms.

In previous work, we reported the development of full-length enriched Arabidopsis cDNA libraries from plants grown under different conditions [13,26] using the biotinylated CAP trapper method with trehalose-thermoactivated reverse transcriptase [27-30]. A total of 155 144 RIKEN Arabidopsis Full-Length (RAFL) clones were isolated and clustered into 14 668 non-redundant cDNA groups [14]. Using the full-length cDNAs, we also created a microarray to analyze the expression profiles of Arabidopsis genes under various stress conditions or in various mutants and transgenic plants [12,26]. Using ectopic expression of full-length cDNAs, a novel gain-of-function system, termed the "FOX hunting system" (Full-length cDNA Over-eXpressing gene hunting system) was developed [31]. The Arabidopsis genome sequence and resources, including full-length cDNAs, also provide powerful tools for comparative genomics in furthering the understanding of the biology and evolution of other plant species [2,5,10,32]. In the present study, we constructed a full-length enriched cDNA library from whole *Thellungiella *plants and various tissues, in addition to cDNAs from seedlings subjected to high salinity, chilling, or freezing stress or to abscisic acid (ABA) treatment. We determined their DNA sequences from both the 5'- and the 3'-ends to permit the functional annotation of the *Thellungiella *full-length cDNAs, and we discuss their predicted functions related to abiotic stress tolerance.

## Results and Discussion

### Full-length enriched cDNA library construction and sequencing of 20 000 cDNAs

We used the biotinylated CAP trapper method [29] to construct a full-length cDNA library of *Thellungiella halophila *(Shandong ecotype) from whole plants as well as from various tissues, including leaves, roots, flowers, siliques, and mature seeds, of plants treated with high salinity, chilling, freezing stress, and ABA (Table 1). The λFLCIII vector [33], which accommodates cDNAs in a broad range of sizes and is useful for the high-efficiency cloning of long cDNA fragments, was used for the construction of the cDNA library. To reduce the frequency of representation of highly expressed mRNAs in the library, normalization procedures [29] were employed in the construction process. The 20000 recombinant clones were randomly selected and sequenced from both ends. We determined 18636 and 16535 sequences from the forward and reverse directions, respectively, and from among the 20000 clones we obtained the forward or reverse sequences of a total of 19429 clones (Table 2). A total of 35171 sequences have been deposited in the DDBJ public sequence database (accession numbers, BY800476 to BY835646). We have named these "RTFL" (RIKEN *Thellungiella *Full-Length) cDNAs.

**Table 1:** Collection of RNA sample for constructing a *Thellungiella *full-length cDNA library

Sample name	Condition	Time course	Condition	Tissues
salt stress	NaCl, 250 mM	1, 2, 3, 7 and 14 day	agar medium	whole plants
cold stress	4°C	2, 4, 8 and 24 hour	soil	rosette leaves
freezing stress	-6°C	1, 2, 4 and 8 hour	soil	rosette leaves
ABA	ABA 50 μM	1, 2, 4 and 8 hour	agar medium	whole plants
various tissues	normal condition		soil	siliques, mature seeds

**Table 2:** Characteristics of full-length *Thellungiella *cDNA library

Source of cDNA	Total no. clones	No. forward sequences	No. reverse sequences	Total no. sequences	Total no. singletons after CAP3 analysis	Total no. contigs after CAP3 analysis	No. gene clusters
RTFL^a^	19429	18636	16535	35171	6556	7402	9569

aRTFL, RIKEN Thellungiella halophila full-length cDNA

**Figure 1:**
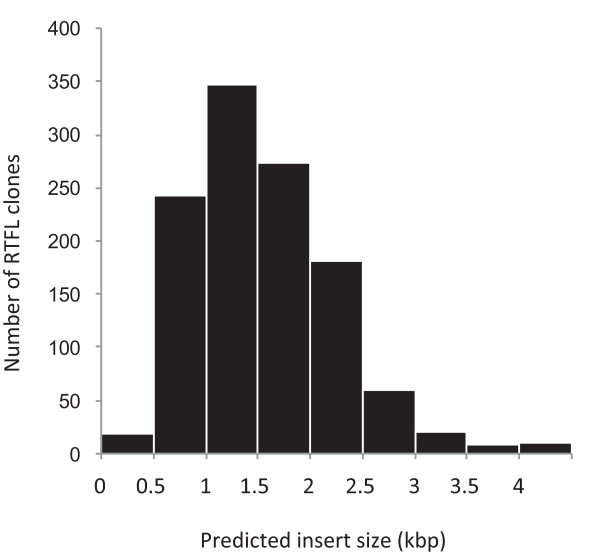
**Size distribution of the RTFL clones**. The sequence lengths of the *Thellungiella *cDNA inserts were determined from a total of 1161 clones by digestion with *SfiI *(in the cDNA cloning site) or PCR amplification using T3 and T7 primers.

Figure 1 shows the size distribution of the *Thellungiella *cDNA inserts from 1161 randomly selected clones. The average size was approximately 1.54 kbp. Our group previously determined 20683 full-read cDNA sequences from the RAFL (RIKEN Arabidopsis Full-Length) cDNA collection, and these sequences are available in the RARGE database [34]. The estimated average size of the RAFL cDNA inserts was 1.495 kbp (Motoaki Seki et al., RIKEN Plant Science Center, unpublished results). The average size of the *Thellungiella *cDNA inserts was thus slightly longer than the average cDNA inserts from Arabidopsis libraries and similar to those in other plants; for example, the average rice and wheat cDNA lengths are both about 1.5 kbp [10,18].

### Sequence assembly and the proportion of full-length cDNA clones in the library

**Figure 2:**
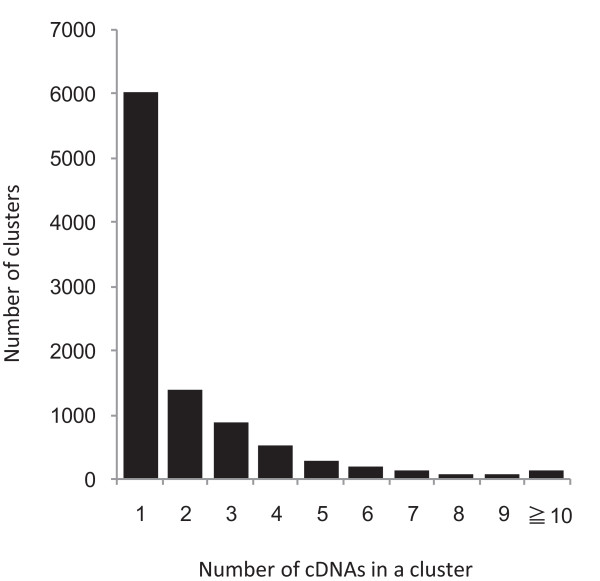
**Sequence redundancy in the normalized cDNA library**. A total of 35171 sequences were assembled and divided into 9569 clusters. The graph represents the number of clones per assembled scaffold. Clusters containing a single cDNA accounted for 63% of the identified sequences, whereas clusters that contained more than six cDNAs accounted for only 5% of the total.

The 35171 sequences were assembled by using the CAP3 program [35] to evaluate the level of sequence redundancy. Assembling these sequences generated 7402 contigs and 6556 singletons, and the sequenced 20000 cDNAs were clustered into 9569 nonredundant scaffolds that represented distinct genes (Table 2). Figure 2 shows the degree of redundancy in the sequences from the cDNA library. The majority of the cDNAs (6024 cDNAs, 63% of the total), consisted of a single cDNA in a cluster, and only 5% contained more than six cDNAs in a cluster, indicating that the normalization procedures were successful.

The cDNA sequence data were submitted for the BLASTN search to compare with the green plants (Viridiplantae) mRNA databases in GenBank. Of the 19429 clones that we obtained as clean sequences, 18 295 clones (94%) showed > 80% identity, whereas the remaining clones (6%) had no significant identity to any plant sequences in GenBank.

We examined the proportion of full-length cDNA clones in our library. We selected clones that (1) had sequences from both ends, and (2) showed an Expected Value (*E*) < 1.0e^-20 ^on the basis of a fastx search using forward sequences as queries against Arabidopsis proteins (TIGR v5, ATH1.pep, ), with the correct direction of the reading frame. We considered clones to be full-length if they met the following criteria: (1) they contained the first methionine, and (2) the reverse sequence contained the polyA sequence. Consequently, we selected 12878 clones as calculation objects and classified 10880 (84.5%) of these clones as full-length clones. This frequency is nearly identical to the reported values from the Arabidopsis [14], rice [15], and wheat [18] libraries.

### Functional annotation of RTFL cDNAs

The 9569 nonredundant genes were submitted to InterPro [36] to obtain functional domain information. InterPro is an integrated resource for protein families, domains and functional sites that integrates the following protein signature databases: PROSITE, PRINTS, ProDom, Pfam, SMART, TIGRFAMs, PIRSF, SUPERFAMILY, Gene3D, and PANTHER. Protein matches in InterPro are pre-calculated by using InterProScan software, which combines the different protein signature recognition methods offered by the InterPro member databases into one resource and provides the corresponding InterPro accession numbers and Gene Ontology (GO) annotations [37]. A total of 8289 sequences were assigned to InterPro IDs and GO terms [see Additional file 1]. According to the obtained GO terms, the 8289 genes were remapped and classified by using Plant GO Slim (; [38]).

**Figure 3:**
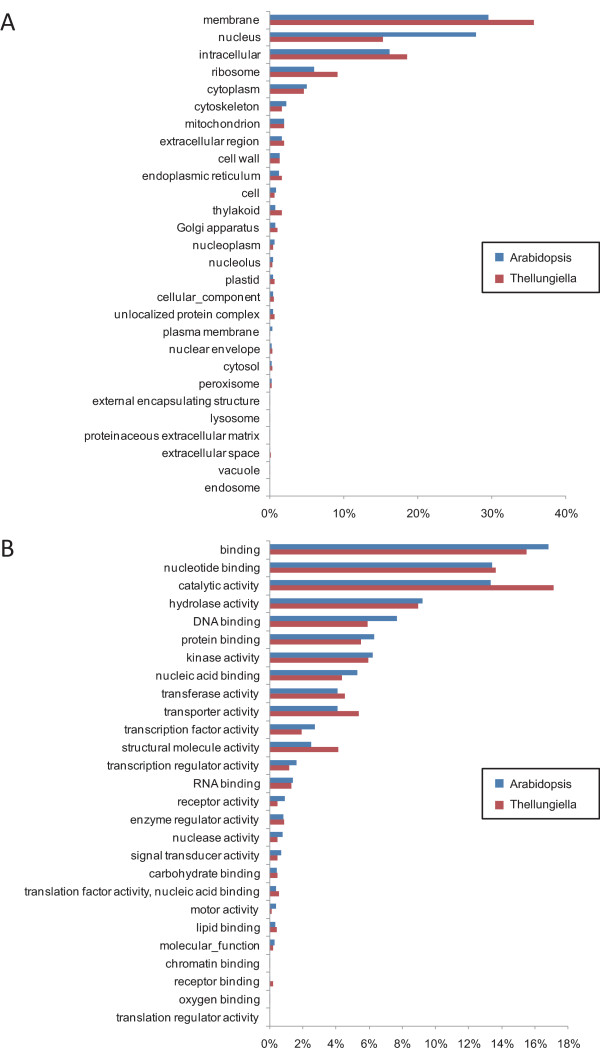
**Comparison of the categories of Arabidopsis and nonredundant *Thellungiella *genes**. The 28227 Arabidopsis genes and the 8298 nonredundant *Thellungiella *genes that were assigned InterPro IDs were classified according to the GO terms using Plant GO Slim  into categories based on (A) cellular components and (B) molecular function.

**Figure 4:**
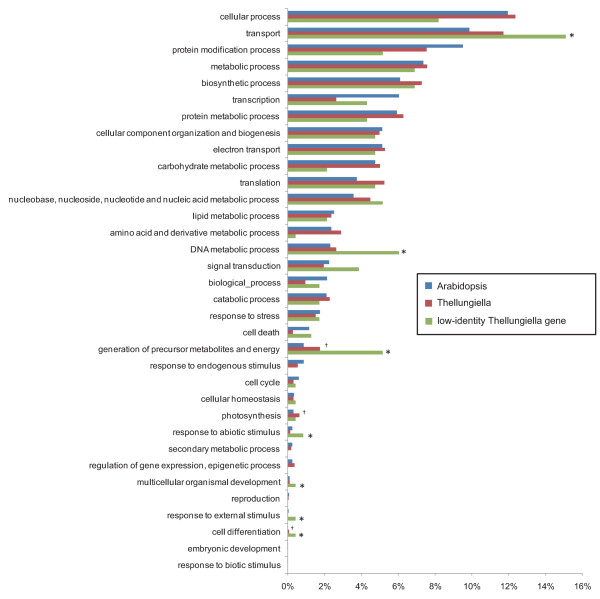
**Biological process categories for Arabidopsis genes, nonredundant *Thellungiella *genes, and *Thellungiella *genes that showed low identity to Arabidopsis genes**. The 28227 Arabidopsis genes, the 8298 nonredundant *Thellungiella *genes, and the 763 *Thellungiella *genes that showed low identity to Arabidopsis genes assigned to InterPro IDs were classified according to the GO terms using Plant GO Slim  for biological processes. † indicates the categories in which the number of *Thellungiella *genes was more than 1.5 times the number of Arabidopsis genes. * indicates categories in which the number of *Thellungiella *genes was more than 1.5 times the number of Arabidopsis genes. The number of *Thellungiella *genes under the categories of transport, DNA metabolic process, generation of precursor metabolites and energy, response to abiotic stimulus, multicellular organismal development, response to external stimulus, and cell differentiation was more than 1.5 times that in Arabidopsis.

Figures 3 and 4 show the categorization of Arabidopsis genes and the 8289 *Thellungiella *genes assigned to the GO terms. In most categories, we observed no obvious differences between the numbers of sequences from Arabidopsis and *Thellungiella*, including genes involved in biological processes, cellular components, and molecular function. Notably, the number of sequences classified as transcription genes under biological processes in Arabidopsis was approximately twice that in *Thellungiella *(Fig. 4). Furthermore, the number of Arabidopsis genes associated with the nucleus under cellular components was also much higher than that in *Thellungiella *(Fig. 3A). Although the total number of transcription factors is not clear in *Thellungiella *genome, there seems to be no significant difference in the proportion of transcription factor genes between Arabidopsis and *Thellungiella *genome. The population of cDNAs may reflects the levels of gene expression. Thus, the expression level of *Thellungiella *transcription factors may be lower than that of Arabidopsis. Arabidopsis responds strongly to abiotic and biotic stresses at the transcriptional level. In contrast, *Thellungiella *does not initiate immediate changes in transcription in response to abiotic stresses, and instead constitutively expresses a large number of genes that correspond to stress-inducible genes in Arabidopsis [5]. Thus, the difference between Arabidopsis and *Thellungiella *in their responses to various stimuli at the transcriptional level may reflect differences in the number of transcription-related genes between the organisms and may depend partly on the number of transcription factors.

### Features of Thellungiella-specific genes

Most *Thellungiella *genes have a high sequence identity (approximately 90% at the cDNA level) to Arabidopsis genes. Numerous studies of salt tolerance in Arabidopsis suggest that this plant contains most, if not all, the salt-tolerance related genes that might be found in halophytes[39]. The current hypothesis is that halophytes employ salt-tolerance mechanisms similar to those found in glycophytes, including Arabidopsis. However, subtle differences in this regulation result in large variations in salt tolerance between glycophytes and halophytes[11]. In addition, halophytes are hypothesized to exhibit specific salt-tolerance mechanisms resulting from the induction of halophyte-specific genes. We divided the 8298 genes into two groups on the basis of their sequence identities, using BLASTX searches against the Arabidopsis database. The group with high sequence identity to Arabidopsis genes (*E *value ≤ 1.0e^-50^) included 7535 genes, and the group with low identity to Arabidopsis genes (*E *value > 1.0e^-50^) included 763 genes. Previous studies revealed that a plasma membrane Na^+^/H^+ ^antiporter (SOS1), a vacuolar Na^+^/H^+ ^antiporter (NHX1), and a plasma membrane Na^+ ^transporter (HKT1) are essential for the salt tolerance of Arabidopsis [40-42], and these mutants exhibit a salt-hypersensitive phenotype. In contrast, plants that overexpress *SOS1 *and *NHX1 *show higher salt tolerance than wild-type plants [43,44]. The co-ortholog *Thellungiella *genes belong to the first gene group, exhibiting high identity to Arabidopsis genes. This suggests that some salt-tolerance mechanisms are common to both glycophytes and halophytes.

We compared the categorization of the whole Arabidopsis genome with the categories of the 763 *Thellungiella *genes that exhibited low identity to Arabidopsis genes (Fig. 4). Of the genes involved in biological processes, the number of genes in the categories for transport, DNA metabolic process, generation of precursor metabolites and energy, response to abiotic stimulus, multicellular organismal development, response to external stimulus, and cell differentiation in *Thellungiella *were more than 1.5 times the number in Arabidopsis (Fig. 4). Moreover, in regards to molecular function, the proportion of genes involved in transporter activity in *Thellungiella *was also higher than in Arabidopsis. Less NaCl accumulates in *Thellungiella *plants than in Arabidopsis under similar salinity conditions, suggesting that *Thellungiella *has a superior system for suppressing Na^+ ^influx or for excreting Na^+ ^[5]. Electrophysiological analysis indicates that *Thellungiella *also exhibits high potassium/sodium selectivity, implying that *Thellungiella *has specific ion channel features that lead to superior homeostasis with respect to sodium and potassium [7]. Arabidopsis that overexpresses a plasma membrane Na^+^/H^+ ^antiporter gene, *SOS1*, shows salinity tolerance and represses its sodium uptake compared with that of wild-type plants [44]. Likewise, the expression level of *SOS1 *in *Thellungiella *is higher than in Arabidopsis [5,45]. Although *SOS1 *overexpression suggests a contribution of this gene to the salt tolerance of *Thellungiella*, the large proportion of transport genes may imply that *Thellungiella *has a distinct ion transportation system regulated by these specific genes.

### Salt tolerance system using Thellungiella-specific transporter genes

Table 3 [see Additional file 2] lists the *Thellungiella *genes with low identity (*E *value > 1.0e^-50^) to the Arabidopsis genes classified under transporter genes. Several transporters, including chloride channels and P-type H^+^-ATPase, play important roles in the salt tolerance of plants. Homeostasis of Na^+ ^and Cl^- ^is an important mechanism to reduce NaCl stress in higher plants. Chloride channels (CLCs) are a group of voltage-gated Cl^- ^channels originally identified in animals [46]; they have diverse cellular functions such as stabilizing cell membrane potential and regulating cell volume and transcellular chloride transport [47]. Recently, a chloride channel gene, *GmCLC1*, was cloned from soybeans [48]. Transgenic tobacco BY-2 cells expressing *GmCLC1 *were able to drain Cl^- ^more efficiently from vacuoles than was the case in untransformed BY-2 cells, and the transgenics showed a higher NaCl tolerance [48]. The plant cell membrane is energized by an electrochemical gradient produced by P-type H^+^-ATPase (proton pump). These pumps are encoded by at least 12 genes in Arabidopsis. One of the Arabidopsis P-type H^+^-ATPase genes, *AHA4*, was expressed most strongly in the root endodermis [49]. The *aha4 *mutant plants exhibited a clear growth reduction under a mild stress of 75 mM NaCl compared with wild-type plants, and the ratio of Na^+ ^to K^+ ^in the *aha4 *mutants increased to between four and five times the values in wild-type plants. These results suggest that the *aha4 *mutants were compromised in their ability to exclude Na^+ ^under salinity stress [49]. P-type H^+^-ATPases were also found in a halotolerant cyanobacterium, *Aphanothece halophytica*, and a marine alga, *Tetraselmis viridis *[50,51]. *Aphanothece halophytica *grows under a wide range of salinity conditions (from 0.25 to 3.0 M NaCl), and Na^+^/H^+ ^antiporters in *A. halophytica *play a crucial role in Na^+ ^efflux to provide enhanced salt tolerance. Since the efflux of Na^+ ^mediated by Na^+^/H^+ ^antiporters utilizes protons as the motive force provided by a primary proton pump, H^+^-ATPase, the P-type H^+^-ATPase is thought to contribute to the salt tolerance of this species [52]. On the other hand, vacuolar ATPase (V-ATPase) is the major proton pump that establishes and maintains an electrochemical proton gradient across the tonoplast. Expression of several V-ATPase subunits or an increase in V-ATPase activity induced by salt stress has been observed in a number of glycophytic species [53], suggesting that increased V-ATPase levels or activity are required to drive Na^+ ^sequestration under salt stress. Recently, the V-ATPase-deficient *det3 *Arabidopsis mutant was shown to be extremely salt sensitive. Moreover, SOS2, a protein kinase that phosphorylates SOS1, interacted directly with the V-ATPase regulatory subunits B1 and B2 [54]. These studies indicate that V-ATPase activity plays a key role in salt tolerance. Although most *Thellungiella *genes show approximately 90% identity with Arabidopsis genes, the *Thellungiella *genes encoding transporters appear to be remarkably different from their Arabidopsis co-orthologs. Whether the sequence diversities among these genes are the source of the large differences in salt tolerance between *Thellungiella *and Arabidopsis is a topic of great interest.

**Table 3:** *Thellungiela *genes showing low identity against Arabidopsis genes classified in 'transport' using GO slim^a^

Clone name	InterPro ID	Description	AGI code^b^	E value c
RTFL01-07-H15	IPR001807	Chloride channel, voltage gated	AT5G40890.2	1.00E-49

RTFL01-12-M19	IPR000194	ATPase, F1/V1/A1 complex, alpha/beta subunit, nucleotide-binding	AT1G60190.1	6.00E-45

RTFL01-29-P17	IPR000463	Cytosolic fatty-acid binding	AT2G25590.1	8.00E-45

RTFL01-05-O18	IPR000803	Facilitated glucose transporter	AT3G58130.2	5.00E-43

RTFL01-21-M15	IPR003612	Plant lipid transfer protein/seed storage/trypsin-alpha amylase inhibitor	AT2G38540.1	5.00E-43

RTFL01-24-G14	IPR000264	Serum albumin	AT5G09460.1	5.00E-43

RTFL01-49-P05	IPR003612	Plant lipid transfer protein/seed storage/trypsin-alpha amylase inhibitor	AT2G38540.1	5.00E-43

RTFL01-36-E03	IPR007271	Nucleotide-sugar transporter	AT5G65000.2	3.00E-41

RTFL01-05-G14	IPR001993	Mitochondrial substrate carrier	AT5G42130.1	3.00E-38

RTFL01-14-G02	IPR000194	ATPase, F1/V1/A1 complex, alpha/beta subunit, nucleotide-binding	AT1G08010.2	1.00E-34

RTFL01-43-C04	IPR002075	Nuclear transport factor 2	AT1G69250.1	4.00E-34

RTFL01-21-H04	IPR004240	Nonaspanin (TM9SF)	AT4G12650.1	5.00E-34

RTFL01-18-D17	IPR006455	Homeobox domain, ZF-HD class	AT5G42780.1	2.00E-31

RTFL01-07-P02	IPR005829	Sugar transporter superfamily	AT4G10410.1	2.00E-30

RTFL01-08-J20	IPR001757	ATPase, P-type, K/Mg/Cd/Cu/Zn/Na/Ca/Na/H-transporter	AT2G31150.1	3.00E-28

RTFL01-06-N05	IPR003612	Plant lipid transfer protein/seed storage/trypsin-alpha amylase inhibitor	AT3G18840.2	1.00E-27

RTFL01-40-M18	IPR004240	Nonaspanin (TM9SF)	AT1G10950.1	1.00E-27

RTFL01-11-J21	IPR000194	ATPase, F1/V1/A1 complex, alpha/beta subunit, nucleotide-binding	AT3G24503.1	7.00E-27

RTFL01-39-I23	IPR008389	ATPase, V0 complex, subunit H	AT4G26710.2	2.00E-23

RTFL01-52-J14	IPR000194	ATPase, F1/V1/A1 complex, alpha/beta subunit, nucleotide-binding	AT3G54760.1	5.00E-21

RTFL01-33-P14	IPR000568	ATPase, F0 complex, subunit A	AT4G13740.1	6.00E-15

RTFL01-01-D06	IPR000194	ATPase, F1/V1/A1 complex, alpha/beta subunit, nucleotide-binding	AT1G29760.1	6.00E-15

RTFL01-20-A07	IPR000194	ATPase, F1/V1/A1 complex, alpha/beta subunit, nucleotide-binding	AT1G29760.1	6.00E-15

RTFL01-28-P24	IPR001622	Voltage-dependent potassium channel	AT5G55430.1	3.00E-13

RTFL01-25-D12	IPR000245	ATPase, V0 complex, proteolipid subunit C,	AT1G75630.1	7.00E-08

RTFL01-22-P12	IPR000264	Serum albumin	AT5G09460.1	1.00E-07

RTFL01-03-P24	IPR006121	Heavy metal transport/detoxification protein	AT5G11890.1	2.00E-06

RTFL01-40-P02	IPR002946	Intracellular chloride channel	AT5G08450.3	3.00E-04

RTFL01-03-G04	IPR003663	Sugar transporter	AT5G50540.1	0.001

RTFL01-11-N06	IPR000109	TGF-beta receptor, type I/II extracellular region	AT3G55610.1	0.019

RTFL01-13-J08	IPR005829	Sugar transporter superfamily	AT5G49665.1	0.073

RTFL01-11-G05	IPR007114	Major facilitator superfamily	AT1G05300.2	0.075

RTFL01-17-J21	IPR000194	ATPase, F1/V1/A1 complex, alpha/beta subunit, nucleotide-binding	AT4G19830.1	0.25

RTFL01-38-L07	IPR011116	SecA Wing and Scaffold	AT3G55160.1	0.76

RTFL01-20-G20	IPR004100	ATPase, F1/V1/A1 complex, alpha/beta subunit, N-terminal	AT5G60470.1	1.2

a The 9,569 nonredundant Thellungiella genes were submitted to InterPro. The 763 Thellungiella genes exhibiting low identity (E value > 1.0e-50) against Arabidopsis genes assigned to InterPro ID were classified according to the GO terms using GO slim.

b Arabidopsis gene showing the highest identity with the Thellungiella cDNA clone.

c Sequence identity between the Thellungiella clone and the Arabidopsis counterpart (show AGI code) using BLASTX searches

against Arabidopsis database.

## Conclusion and cDNA resources

We generated a full-length enriched cDNA library of *Thellungiella halophila *from various tissues and whole plants treated with salinity, chilling, freezing stresses, or ABA. We isolated about 20000 full-length enriched cDNA clones (RTFL cDNAs) and sequenced them from both ends, and we outlined the features of their predicted functions (coding *Thellungiella *proteins) by comparing them with those of Arabidopsis. Moreover, the 35171 RTFL cDNA sequences have been deposited in the DDBJ public data center. The number of *T. halophila *(*T. salsuginea*) ESTs entries was 9388 as of July 2008, which means that our effort has increased the number of ESTs by four times the number before our study. Our sequences will thus contribute to correct annotation of the *Thellungiella *genome sequence in the near future. The RTFL cDNA clones will also enable the construction of overexpressing mutant plants by introduction of the cDNAs driven by a constitutive promoter, as well as the complementation of *Thellungiella *mutants and the determination of promoter regions in the *Thellungiella *genome. The RTFL clones will be available for distribution through the RIKEN Bioresource Center .

## Methods

### Plant materials and stress treatments

*Thellungiella halophila *(Shandong ecotype) seeds were sown on Murashige and Skoog (MS) plates containing 0.8% (wt/vol) agar and 1% sucrose. The seeds were stratified at 4°C for two weeks and then transferred to 22°C under continuous light for germination and growth. Three weeks after germination, seedlings of *Thellungiella *were transferred to 250 mM NaCl (salt stress) or 50 μM ABA (ABA treatment) water, or were transferred onto separate 9-cm plastic pots filled with a 1:1 mixture of perlite/vermiculite and watered with 1000-fold diluted Hyponex™ (Hyponex, Osaka, Japan). One week after transfer onto the soil pots under 16 hours light – 8 hours darkness at 22°C, the seedlings were subjected to 4 °C (cold stress) or -6°C (freezing stress) in a growth chamber under 24 hours darkness.

A *Thellungiella *full-length cDNA library was constructed from a mixture of mRNA extracted from stress-treated plants and various tissues of *Thellungiella*. *Thellungiella *plants were subjected to various stress treatments: high-salinity (250 mM NaCl for 1, 2, 3, 7, and 14 days), cold temperatures (4°C for 2, 4, 8, and 24 hours), freezing temperatures (-6°C for 1, 2, 4, or 8 hours), or ABA (50 μM ABA for 1, 2, 4, and 8 hours). Control plants were grown under unstressed conditions under16 hours light – 8 hours darkness at 22°C. After the stress treatments, mRNA was extracted from whole plants (salt stress and ABA treatment) or rosette leaves (cold and freezing stresses) collected at each point in time. Rosette leaves and cauline leaves, roots, flowers, and siliques were collected from 7- to 10-week-old plants, and mature seeds were collected from 12- to 20-week-old plants.

### RNA extraction and construction of a full-length cDNA library

Total RNA was prepared by using TRIZOL Reagent (Life Technologies, Rockville, MD, USA) from the treated samples. A full-length cDNA library was constructed as previously reported [14,27,28] by means of the biotinylated CAP trapper method using trehalose-thermoactivated reverse transcriptase [28]. We used the λFLCIII [33] vector, which accommodates cDNAs in a broad range of sizes and is thus useful for the high-efficiency cloning of long cDNA fragments, for construction of the cDNA libraries [33]. The λFLCIII vectors can also be bulk-excised by a Cre-*lox*-based system free of size bias to generate the plasmid libraries. Normalization [29] was also introduced in the construction of the full-length cDNA library to reduce the frequency of highly expressed mRNAs in the library. The method is based on hybridization of first-strand, full-length cDNA as the tester and cellular biotinylated RNA extracted from stress-treated plants and various tissues of *Thellungiella *as the normalizing driver.

### Sequencing of *Thellungiella *cDNA clones

The DNA of each clone was directly amplified from 384 bacterial cultures in a glycerol stock plate by the RCA method [55] using a TempliPhi HT DNA amplification kit (GE Healthcare, United Kingdom). End sequencing of 20000 clones was performed using ABI 3700 capillary sequencers (Applied Biosystems, Foster City, CA, U.S.). The M13 (-21) primer (5'-TGTAAAACGACGGCCAGT-3') and the 1233 primer (5'-AGCGGATAACAATTTCACACAGGA-3') were used for forward and reverse sequencing, respectively.

### Trimming of sequence data and assembly

We used sim4 software for the detection of vector sequences [56]. Raw sequence data were base-called using the Phred software [57,58]. Regions of low quality found at both edges of each raw sequence were discarded, and we extracted only the high-quality region (Phred quality score > 14, and more than 20 bases repeated). After this initial evaluation, sequence data shorter than 100 bases in length or with many low-quality regions (Phred quality score ≤ 14, and more than 50% of its total length) were omitted. The ESTs were assembled by using CAP3 software [35] with its default parameters. All sequences were submitted to the DNA Databank of Japan (DDBJ) with accession numbers BY800476 to BY835646.

### cDNA insert size of the RTFL clones

The sequence lengths of the *Thellungiella *cDNA inserts were determined from a total of 1,161 clones by digestion with *SfiI *(in the cDNA cloning site) or PCR amplification using T3 (5'-TGTAAAACGACGGCCAGT-3') and T7 primers (5'-AATACGACTCACTATAGGG-3').

### Full-length cDNA library quality

To examine the proportion of full-length cDNA clones in this library, we selected the following clones as calculation objects: (1) clones with sequences from both ends, and (2) clones showing an Expected Value of (*E*) < 1.0e^-20 ^in a fastx search using forward sequences as queries against Arabidopsis proteins (TIGR v5, ATH1.pep), with the correct direction of the reading frame. We used the following criteria to classify clones as full-length: (1) clones must include the first methionine, and (2) the reverse reading sequence must include the polyA sequence.

### Scaffold construction

In order to obtain a non-redundant set of transcripts, the clones were clustered according to clone names. To accomplish this, we parsed the .ace file from the CAP3 program output to build scaffolds, which are groups of sequences that represent a unique transcript for which the relative position and orientation of the fragments can be inferred. Using clone names, the contigs or singletons corresponding to the two ends of a given clone were joined by adding 20 N's in the middle of both sequences. Since 20 is more than the default window size in BLAST searches, these N's did not interfere with the BLAST analyses.

### Functional annotation of the sequences

Once these scaffolds were created, the sequences were submitted to InterPro [36] to obtain functional domain information. Protein matches in InterPro were pre-calculated with InterProScan software, available from [37,59]. InterProScan provided the corresponding InterPro accession numbers and GO annotation in the results [37]. The genes assigned to InterPro ID were classified according to the GO terms developed by InterPro using Plant GO Slim (; [38]).

## Authors' contributions

TT contributed to and participated in the entire study and drafted the manuscript. TS, KM, AI, and AK performed the bioinformatics analyses (assembly, clustering, annotation and comparative analysis). YT carried out annotation and registration in DDBJ. AT and YS conducted sequencing of the cDNA clones. MS assisted in the construction of the cDNA library. HO checked the length distribution of the cDNA inserts. YS and ST helped draft the manuscript. KS coordinated the project and helped draft the manuscript.

## Supplementary Material

Additional file 1 **List of 9,569 culusters with accession numbers and the annotation.** Click here for file

Additional file 2 **Thellungiela genes showing low identity against Arabidopsis genes classified in 'transport' using GO slim with the most homologous gene.** Click here for file

## References

[B1] Bressan RA, Zhang C, Zhang H, Hasegawa PM, Bohnert HJ, Zhu JK (2001). Learning from the Arabidopsis experience. The next gene search paradigm. Plant Physiol.

[B2] Gong Q, Li P, Ma S, Indu Rupassara S, Bohnert HJ (2005). Salinity stress adaptation competence in the extremophile Thellungiella halophila in comparison with its relative Arabidopsis thaliana. Plant J.

[B3] Inan G, Zhang Q, Li P, Wang Z, Cao Z, Zhang H, Zhang C, Quist TM, Goodwin SM, Zhu J (2004). Salt cress. A halophyte and cryophyte *Arabidopsis *relative model system and its applicability to molecular genetic analyses of growth and development of extremophiles. Plant Physiol.

[B4] Li P, Mane SP, Sioson AA, Robinet CV, Heath LS, Bohnert HJ, Grene R (2006). Effects of chronic ozone exposure on gene expression in Arabidopsis thaliana ecotypes and in Thellungiella halophila. Plant Cell Environ.

[B5] Taji T, Seki M, Satou M, Sakurai T, Kobayashi M, Ishiyama K, Narusaka Y, Narusaka M, Zhu JK, Shinozaki K (2004). Comparative genomics in salt tolerance between Arabidopsis and Arabidopsis-related halophyte salt cress using Arabidopsis microarray. Plant Physiol.

[B6] Vera-Estrella R, Barkla BJ, Garcia-Ramirez L, Pantoja O (2005). Salt stress in Thellungiella halophila activates Na+ transport mechanisms required for salinity tolerance. Plant Physiol.

[B7] Volkov V, Amtmann A (2006). Thellungiella halophila, a salt-tolerant relative of Arabidopsis thaliana, has specific root ion-channel features supporting K+/Na+ homeostasis under salinity stress. Plant J.

[B8] Wong CE, Li Y, Labbe A, Guevara D, Nuin P, Whitty B, Diaz C, Golding GB, Gray GR, Weretilnyk EA (2006). Transcriptional profiling implicates novel interactions between abiotic stress and hormonal responses in Thellungiella, a close relative of Arabidopsis. Plant Physiol.

[B9] Wong CE, Li Y, Whitty BR, Diaz-Camino C, Akhter SR, Brandle JE, Golding GB, Weretilnyk EA, Moffatt BA, Griffith M (2005). Expressed sequence tags from the Yukon ecotype of Thellungiella reveal that gene expression in response to cold, drought and salinity shows little overlap. Plant Mol Biol.

[B10] Alexandrov NN, Troukhan ME, Brover VV, Tatarinova T, Flavell RB, Feldmann KA (2006). Features of Arabidopsis genes and genome discovered using full-length cDNAs. Plant Mol Biol.

[B11] Zhu JK (2001). Plant salt tolerance. Trends Plant Sci.

[B12] Seki M, Satou M, Sakurai T, Akiyama K, Iida K, Ishida J, Nakajima M, Enju A, Narusaka M, Fujita M (2004). RIKEN Arabidopsis full-length (RAFL) cDNA and its applications for expression profiling under abiotic stress conditions. J Exp Bot.

[B13] Seki M, Carninci P, Nishiyama Y, Hayashizaki Y, Shinozaki K (1998). High-efficiency cloning of Arabidopsis full-length cDNA by biotinylated CAP trapper. Plant J.

[B14] Seki M, Narusaka M, Kamiya A, Ishida J, Satou M, Sakurai T, Nakajima M, Enju A, Akiyama K, Oono Y (2002). Functional annotation of a full-length Arabidopsis cDNA collection. Science.

[B15] Kikuchi S, Satoh K, Nagata T, Kawagashira N, Doi K, Kishimoto N, Yazaki J, Ishikawa M, Yamada H, Ooka H (2003). Collection, mapping, and annotation of over 28,000 cDNA clones from japonica rice. Science.

[B16] Nanjo T, Futamura N, Nishiguchi M, Igasaki T, Shinozaki K, Shinohara K (2004). Characterization of full-length enriched expressed sequence tags of stress-treated poplar leaves. Plant Cell Physiol.

[B17] Nanjo T, Sakurai T, Totoki Y, Toyoda A, Nishiguchi M, Kado T, Igasaki T, Futamura N, Seki M, Sakaki Y (2007). Functional annotation of 19,841 Populus nigra full-length enriched cDNA clones. BMC Genomics.

[B18] Ogihara Y, Mochida K, Kawaura K, Murai K, Seki M, Kamiya A, Shinozaki K, Carninci P, Hayashizaki Y, Shin IT (2004). Construction of a full-length cDNA library from young spikelets of hexaploid wheat and its characterization by large-scale sequencing of expressed sequence tags. Genes Genet Syst.

[B19] Jia J, Fu J, Zheng J, Zhou X, Huai J, Wang J, Wang M, Zhang Y, Chen X, Zhang J (2006). Annotation and expression profile analysis of 2073 full-length cDNAs from stress-induced maize (Zea mays L.) seedlings. Plant J.

[B20] Ota T, Suzuki Y, Nishikawa T, Otsuki T, Sugiyama T, Irie R, Wakamatsu A, Hayashi K, Sato H, Nagai K (2004). Complete sequencing and characterization of 21,243 full-length human cDNAs. Nat Genet.

[B21] Carninci P, Waki K, Shiraki T, Konno H, Shibata K, Itoh M, Aizawa K, Arakawa T, Ishii Y, Sasaki D (2003). Targeting a complex transcriptome: the construction of the mouse full-length cDNA encyclopedia. Genome Res.

[B22] Kawai J, Shinagawa A, Shibata K, Yoshino M, Itoh M, Ishii Y, Arakawa T, Hara A, Fukunishi Y, Konno H (2001). Functional annotation of a full-length mouse cDNA collection. Nature.

[B23] Rubin GM, Hong L, Brokstein P, Evans-Holm M, Frise E, Stapleton M, Harvey DA (2000). A Drosophila complementary DNA resource. Science.

[B24] Stapleton M, Carlson J, Brokstein P, Yu C, Champe M, George R, Guarin H, Kronmiller B, Pacleb J, Park S (2002). A Drosophila full-length cDNA resource. Genome Biol.

[B25] Stapleton M, Liao G, Brokstein P, Hong L, Carninci P, Shiraki T, Hayashizaki Y, Champe M, Pacleb J, Wan K (2002). The Drosophila gene collection: identification of putative full-length cDNAs for 70% of D. melanogaster genes. Genome Res.

[B26] Seki M, Narusaka M, Ishida J, Nanjo T, Fujita M, Oono Y, Kamiya A, Nakajima M, Enju A, Sakurai T (2002). Monitoring the expression profiles of 7000 Arabidopsis genes under drought, cold and high-salinity stresses using a full-length cDNA microarray. Plant J.

[B27] Carninci P, Kvam C, Kitamura A, Ohsumi T, Okazaki Y, Itoh M, Kamiya M, Shibata K, Sasaki N, Izawa M (1996). High-efficiency full-length cDNA cloning by biotinylated CAP trapper. Genomics.

[B28] Carninci P, Nishiyama Y, Westover A, Itoh M, Nagaoka S, Sasaki N, Okazaki Y, Muramatsu M, Hayashizaki Y (1998). Thermostabilization and thermoactivation of thermolabile enzymes by trehalose and its application for the synthesis of full length cDNA. Proc Natl Acad Sci USA.

[B29] Carninci P, Shibata Y, Hayatsu N, Sugahara Y, Shibata K, Itoh M, Konno H, Okazaki Y, Muramatsu M, Hayashizaki Y (2000). Normalization and subtraction of cap-trapper-selected cDNAs to prepare full-length cDNA libraries for rapid discovery of new genes. Genome Res.

[B30] Carninci P, Westover A, Nishiyama Y, Ohsumi T, Itoh M, Nagaoka S, Sasaki N, Okazaki Y, Muramatsu M, Schneider C (1997). High efficiency selection of full-length cDNA by improved biotinylated cap trapper. DNA Res.

[B31] Ichikawa T, Nakazawa M, Kawashima M, Iizumi H, Kuroda H, Kondou Y, Tsuhara Y, Suzuki K, Ishikawa A, Seki M (2006). The FOX hunting system: an alternative gain-of-function gene hunting technique. Plant J.

[B32] Nishiyama T, Fujita T, Shin IT, Seki M, Nishide H, Uchiyama I, Kamiya A, Carninci P, Hayashizaki Y, Shinozaki K (2003). Comparative genomics of Physcomitrella patens gametophytic transcriptome and Arabidopsis thaliana: implication for land plant evolution. Proc Natl Acad Sci USA.

[B33] Carninci P, Shibata Y, Hayatsu N, Itoh M, Shiraki T, Hirozane T, Watahiki A, Shibata K, Konno H, Muramatsu M (2001). Balanced-size and long-size cloning of full-length, cap-trapped cDNAs into vectors of the novel lambda-FLC family allows enhanced gene discovery rate and functional analysis. Genomics.

[B34] Sakurai T, Satou M, Akiyama K, Iida K, Seki M, Kuromori T, Ito T, Konagaya A, Toyoda T, Shinozaki K (2005). RARGE: a large-scale database of RIKEN Arabidopsis resources ranging from transcriptome to phenome. Nucleic Acids Res.

[B35] Huang X, Madan A (1999). CAP3: A DNA sequence assembly program. Genome Res.

[B36] Zdobnov EM, Apweiler R (2001). InterProScan – an integration platform for the signature-recognition methods in InterPro. Bioinformatics.

[B37] Mulder NJ, Apweiler R, Attwood TK, Bairoch A, Bateman A, Binns D, Bork P, Buillard V, Cerutti L, Copley R (2007). New developments in the InterPro database. Nucleic Acids Res.

[B38] Ashburner M, Ball CA, Blake JA, Botstein D, Butler H, Cherry JM, Davis AP, Dolinski K, Dwight SS, Eppig JT (2000). Gene ontology: tool for the unification of biology. The Gene Ontology Consortium. Nat Genet.

[B39] Zhu JK (2000). Genetic analysis of plant salt tolerance using Arabidopsis. Plant Physiol.

[B40] Apse MP, Sottosanto JB, Blumwald E (2003). Vacuolar cation/H+ exchange, ion homeostasis, and leaf development are altered in a T-DNA insertional mutant of AtNHX1, the Arabidopsis vacuolar Na+/H+ antiporter. Plant J.

[B41] Shi H, Ishitani M, Kim C, Zhu JK (2000). The Arabidopsis thaliana salt tolerance gene SOS1 encodes a putative Na+/H+ antiporter. Proc Natl Acad Sci USA.

[B42] Sunarpi, Horie T, Motoda J, Kubo M, Yang H, Yoda K, Horie R, Chan WY, Leung HY, Hattori K (2005). Enhanced salt tolerance mediated by AtHKT1 transporter-induced Na unloading from xylem vessels to xylem parenchyma cells. Plant J.

[B43] Apse MP, Aharon GS, Snedden WA, Blumwald E (1999). Salt tolerance conferred by overexpression of a vacuolar Na+/H+ antiport in Arabidopsis. Science.

[B44] Shi H, Lee BH, Wu SJ, Zhu JK (2003). Overexpression of a plasma membrane Na+/H+ antiporter gene improves salt tolerance in Arabidopsis thaliana. Nat Biotechnol.

[B45] Kant S, Kant P, Raveh E, Barak S (2006). Evidence that differential gene expression between the halophyte, Thellungiella halophila, and Arabidopsis thaliana is responsible for higher levels of the compatible osmolyte proline and tight control of Na+ uptake in T. halophila. Plant Cell Environ.

[B46] Chen TY (2005). Structure and function of clc channels. Annu Rev Physiol.

[B47] Hechenberger M, Schwappach B, Fischer WN, Frommer WB, Jentsch TJ, Steinmeyer K (1996). A family of putative chloride channels from Arabidopsis and functional complementation of a yeast strain with a CLC gene disruption. J Biol Chem.

[B48] Li WY, Wong FL, Tsai SN, Phang TH, Shao G, Lam HM (2006). Tonoplast-located GmCLC1 and GmNHX1 from soybean enhance NaCl tolerance in transgenic bright yellow (BY)-2 cells. Plant Cell Environ.

[B49] Vitart V, Baxter I, Doerner P, Harper JF (2001). Evidence for a role in growth and salt resistance of a plasma membrane H+-ATPase in the root endodermis. Plant J.

[B50] Popova L, Balnokin Y, Dietz KJ, Gimmler H (1998). Na+-ATPase from the plasma membrane of the marine alga Tetraselmis (Platymonas) viridis forms a phosphorylated intermediate. FEBS Lett.

[B51] Wiangnon K, Raksajit W, Incharoensakdi A (2007). Presence of a Na+-stimulated P-type ATPase in the plasma membrane of the alkaliphilic halotolerant cyanobacterium Aphanothece halophytica. FEMS Microbiol Lett.

[B52] Waditee R, Hibino T, Tanaka Y, Nakamura T, Incharoensakdi A, Takabe T (2001). Halotolerant cyanobacterium Aphanothece halophytica contains an Na(+)/H(+) antiporter, homologous to eukaryotic ones, with novel ion specificity affected by C-terminal tail. J Biol Chem.

[B53] Kirsch M, An Z, Viereck R, Low R, Rausch T (1996). Salt stress induces an increased expression of V-type H(+)-ATPase in mature sugar beet leaves. Plant Mol Biol.

[B54] Batelli G, Verslues PE, Agius F, Qiu Q, Fujii H, Pan S, Schumaker KS, Grillo S, Zhu JK (2007). SOS2 promotes salt tolerance in part by interacting with the vacuolar H+-ATPase and upregulating its transport activity. Mol Cell Biol.

[B55] Dean FB, Nelson JR, Giesler TL, Lasken RS (2001). Rapid amplification of plasmid and phage DNA using Phi 29 DNA polymerase and multiply-primed rolling circle amplification. Genome Res.

[B56] Florea L, Hartzell G, Zhang Z, Rubin GM, Miller W (1998). A computer program for aligning a cDNA sequence with a genomic DNA sequence. Genome Res.

[B57] Ewing B, Green P (1998). Base-calling of automated sequencer traces using phred. II. Error probabilities. Genome Res.

[B58] Ewing B, Hillier L, Wendl MC, Green P (1998). Base-calling of automated sequencer traces using phred. I. Accuracy assessment. Genome Res.

[B59] Petryszak R, Kretschmann E, Wieser D, Apweiler R (2005). The predictive power of the CluSTr database. Bioinformatics.

